# Antipyrine

**Published:** 1888-05

**Authors:** C. A. Brooks

**Affiliations:** Americus, Ga.


### ANTIPYRINE.

BY C. A. BROOKS, M. D., AMERICUS, GA.

No subject appertaining to medicine, and looking to the most useful and practical results, has, perhaps in the past decade of unusual advance in scientific investigation, received more attention than cause and effect of high temperature and the different modes of reducing and controlling it. Nothing in medicine, perhaps, is more universally agreed upon than the fact that the effects of continued high temperature are fatal to organized tissues, and in the highest degree disastrous to the vital forces; hence the importance of thoroughly understanding and appreciating any measure or drug which promises to give us control over this life-destroying element of disease, and I hope to be able to say something of interest to the profession upon Antipyrine, the drug which certainly seems to lead the host of antipyretics which have recently entered the field and begun to clamor for popular favor.

The chemical ccmposition of this preparation, which comes to us under the formidable technical name of *dimethyloxichimzin*, can be of little practical use, and we therefore pass over it, except to give some of its most characteristic appearances and reactions by which it may be recognized. It is a grayish or white bulky crystalline powder, which melts at 113° C., and is easily soluble in water. It gives precipitates with most alkaloidal reagents. Upon treatment with fuming nitric acid, antipyrine becomes green; with carbolic acid, muddy, brownish violet; with salicylic acid, dark yellowish brown. The greenish color of dilute antipyrine solution is permanent for more than a day. When this green solution is heated and treated with a drop of fuming nitric acid, it changes, first to light red, then bluish red.

By way of experiment to determine its physiological effects and its *modus operandi* in disease, I gave to a healthy adult ten grains of the drug dissolved in water. One hour after the administration of the drug, the subject sitting quietly in a comfortable apartment, the face was notably flushed, there was a glowing sensation over the body, and a sense of comfort and well-being experienced, clearly indicating its soothing effect upon the nervous system. The temperature had been reduced from 98½ degrees to 98¼ degrees, and the pulse had fallen from 80 to 70, but not materially reduced in force. At 9 o'clock, three hours after taking, the flush had disappeared from the face, the glowing sensation had subsided, there was a feeling of comfort and quietude as before, while sitting still, but upon exertion there was experienced a feeling of discomfort indicating depression, slight yet clearly perceptible, which prompted the subject to remain quiet. The pulse was found down to 68, and showed very slight loss of force, if any. The temperature was reduced to 98. At 10 o'clock, four hours after taking, the patient retired, feeling more or less drowsy. The pulse was found to be 76, with slight loss of force, and temperature still 98. He reported next morning that he had slept heavier than usual, and awaked a little later than usual, feeling as though he had partaken liberally of onions the night before. This drowsiness, however, was soon shaken off and no unpleasant symptoms followed. It will be noted that there was no sweating produced, and what effect it had on the temperature must have come from its control over heat production, as it did not appear to increase heat dissipation.

The result of this experiment, with my clinical experience with the drug, causes me to place a very high estimate upon Antipyrine as a therapeutic agent, and creates for it a wider range of usefulness than it has already attained. It is gratifying also to know that the position which my personal experience has authorized with regard to it is not unsupported by the most favorable reports which come to us through the prominent journals and from the highest authorities.

IN TYPHOID FEVER.—I have treated five cases of typho-malarial fever in which Antipyrine, with an occasional bath, was relied upon almost exclusively when an antipyretic was required, and in only one case has it failed to produce the desired effect as an antipyretic, and has always acted in a most delightful and salutary manner upon the nervous system, often obviating the use of opium, which, under other treatment, had been with me so essential to the comfort of the patient and to the production of sleep, so necessary to the preservation of the vital forces. It has seemed to me to act especially well in this class of fevers, and in the single instance in which it failed to control the temperature, the patient seemed to tolerate high temperature better, appearing comfortable and not in the least nervous under a temperature of 104½ degrees. Before I began the use of antipyrine, I had relied most largely upon salicylate of soda as an antipyretic, supplemented by an occasional bath. Frequently, under this treatment, I have had the dissatisfaction of seeing my patient's stomach completely upset, the nervous system shattered, nutrition interfered with; then morphia, hypodermically, has been administered to support the patient through this severe trial of his strength, and this, in turn, is followed by a disturbance of all the secretions and other unpleasant after-effects of opiates with which all are familiar. But since beginning the use of Antipyrine all this inconvenience has been avoided. I give it, contrary to the usual mode of administration, in small doses (8 to 10 grains) every three or four hours, beginning in the morning and giving all day in anticipation of the evening exacerbation. I like this better for the reason that I find that if the temperature once gets the upper-hand of me, reaching above 103 degrees, the patient becomes excited, and in order to make an impression such large doses require to be administered that there is too sudden depression, and I fear prostration. But beginning, as I do, with small doses, given, as stated above, in anticipation of the evening exacerbation, I have had the satisfaction of seeing the frailest and most delicate patients pass a comfortable day, the stomach undisturbed, food sometimes (milk, eggs, soup, etc.) taken with evident relish and the temperature remain below 102 degees. This satisfactory condition I have seen day after day throughout an attack lasting from three to seven weeks, followed by a prompt convalescence.

Guttman, of Berlin, 1884, reported upon the action of Antipyrine in thirty-seven cases of febrile disorders. He gave in quantities of one drachm to one and a half drachms in three hourly doses. Under this plan he found the temperature fell from 2½° to 3½° Fahr., the effects lasting five hours and frequently longer. He says "Antipyrine is a powerful and very certain antipyretic." Dr. Francis Minot, Boston, has recently reported (24) twenty-four cases of typhoid fever treated with Antipyrine and Thalline, from which he concludes that all the effects which are claimed for the treatment of typhoid fever by the cold bath are readily obtained without the trouble and inconvenience of the latter method, and without exposing the patient to the dangers of exhaustion, consequent upon the fatigue of removal from bed, and these remedies may be given without danger to the youngest patient.

ANTIPYRINE IN PUERPERAL FEVER.—Dr. Paul F. Munde (New York *Med. Journal*, Oct. 9, 1886) reports 28 cases of puerperal septicæmia and puerperal peritonitis, which he had seen in consultation and treated with Antipyrine, whenever an antipyretic was required, only three of which proved fatal. This certainly is a favorable showing, when we consider that a consultant is not often called until a case becomes very serious, and one of them was actually moribund when he saw her. He says to give it in large doses, say 20 to 30 grains every hour, is hazardous, and entails danger of collapse. He saw the temperature fall from 104° to 96½° Fahr. in a case of pelvic peritonitis within three hours after two doses of 20 grains each. His method of administering the drug is to give 20 grains (if the patient is strong) and to follow it up with 5 or 10 grain doses every half hour or hour until 20 grains more have been taken. If the patient is weak he begins with 10 grain doses, and gives 5 grains every half hour until 20 grains more have been taken. The pulse is carefully watched, and any sign of flagging means discontinuance of Antipyrine and the use of stimulants. As soon as temperature fell below 101 the remedy was discontinued, and begun again as soon as it reached 102, and he seldom gave this quantity, 30 grains, by this plan more than twice in 24 hours, and generally only once, usually toward evening. He never had a case of collapse from its use, and reports nothing but the happiest results. He sometimes gave it hypodermically.

ANTIPYRINE IN RHEUMATISM.—Dr. Jolebiewski, while attached to the military forces in Dresden, has observed the effects of Antipyrine in seventy cases of rheumatism with uniformly good results. He places it on equality with salicylic acid. He gave it in the heroic dose of 60 grains, night and morning, where there was no complication, and the weak state of the patient did not contra-indicate it. There was no case of collapse. The amount of perspiration varied with different individuals. Nausea was quite often observed.—*Va. Med. Monthly.*

"From the date of the first application of antipyrine to theraputics," says T. Mortimer Lloyd in a recent number of *Gaillard's Journal*, "it has been employed in the treatment of rheumatism, and since early in 1884, Alexander has noticed its good effects in calming articular pains. These observations have been confirmed by Dunme, Demuth, Masius, Bernheim, Fraenkel and others."

Dr. Germain See (*Therapeutic Gazette*) says: "In fifteen cases of subacute rheumatism or hydrarthrosis, treated unsuccessfully by salicylate of sodium and ignipuncture, under antipyrine, the swelling and pain disappeared, and the medicine being continued there was no relapse.

At the meeting of the *verein fur innere*, at Berlin, on the 18th November, 1885, Dr. A. Fraenkel reported 34 cases of acute rheumatism treated with antipyrine, given in doses of 15 grammes during the first three days and 3 grammes daily during the next six days. Nine out of thirteen mild cases, and four out of twenty-one severe cases, went on to rapid recovery. In two cases it had no effect and in fifteen there was a relapse. From this he concludes that while Antipyrine is an energetic specific, anti-rheumatic agent, it cannot absolutely replace other rheumatic remedies, some cases reacting to one drug and some to others.

ANTIPYRINE IN PULMONARY DISEASE.—I have treated several cases of pneumonia with the most satisfactory results very recently in which Antipyrine, with an occasional dose of the old reliable, quinine, was all the treatment employed. One case deserves especially to be mentioned in which, owing to the age of the patient and the consequent difficulty of the administration of quinine, antipyrine was the only drug taken after a brisk purgative which had been given before I saw the case. This was a child three years old—was taken with chill, followed by high fever, cough and other symptoms of pneumonia the day before I saw it. When I saw it on second day of the attack, he had a temperature of 104½, extremely nervous, skin dry, cough distressing. I ordered 5 grains antipyrine every 2 hours. Saw him an hour after taking second dose and found nervous symptoms all abated; there was a nice perspiration all over the body, temperature reduced to 101 and the little patient perfectly comfortable. The antipyrine was continued every four hours until 10 o'clock at night. At this time the patient was perfectly quiet, temperature 99½, and inclined to sleep. The medicine was then discontinued until six o'clock in the morning, when it was again begun and kept up through the day every four hours. In anticipation of the evening exacerbation and again discontinued through the night as before, and so on to the seventh day, when the attack terminated by crisis and convalescence was promptly effected. Never before had I had its effect upon nervous reflex action so clearly demonstrated, nor its antipyretic virtues so much appreciated.

Evidence of its good results in controlling high temperature in phthisis comes to us from all sides, and phthisis is placed in the same class with typhoid fevers as being one of the diseases in which the very best results are obtained from its use—a result we would most naturally expect when we consider that the depression following its administration is so slight and that there are no unpleasant after-effects. But the usefulness of the drug in phthisis does not cease here, but we have abundant evidence of its prompt and most satisfactory control of hæmoptisis. Dr. Byvalkevisch reports his results in six cases. In doses of 20 to 80 grains he has arrested hemorrhage where ergot failed.—*American Journal of Medical Sciences.*

Olikhow reports the successful treatment of several cases of hæmoptisis by inhalation of antipyrine. The inhalation employed by him was in the proportion of 4 to 14. The cases thus treated had been under the ordinary management without success.

Friedlander has studied with care the effects of antipyrine in cases of acute bronchitis in children. He divides them in two classes. The first group includes those who have a temperature of 104 degrees, Fahr., and are well nourished; the second, those who have a moderate temperature, 102 degrees, Fahr., or less, and are not well nourished. Both classes are aided by the drug, but the former much more so. Usually, after the administration of a dose, the little patients break out into a profuse perspiration, the temperature falls, they sleep for an hour or so and wake with a feeling of well-being. The dose recommended is: for children under five years old and over two, twelve or thirteen grains about twice daily; to a child two years old, or less, seven grains may be given, but only once in twenty-four hours. This treatment, as recommended by Friedlander, does not include the simultaneous administration of expectorants.

ANTIPYRINE IN NERVOUS DISEASES.—In this class of cases we have good results from the use of antipyrine equal only to its striking results in treatment of hyperpyrexia. In supra-orbital neuralgia, migraine and headaches of all kinds due to nervous reflex causes I have prescribed the drug in doses of from 10 to 25 grains with the most satisfactory results. Its delightful effects have been so apparent and its freedom from unpleasant after-effects so much appreciated by the class of patients who suffer periodically with sick-headache that many of them keep it on hand and take it in doses from 10 to 20 grains at the very beginning of the attack and thus save themselves from the hours of suffering which they had heretofore endured rather than undergo the two or three days of nausea and discomfort which almost invariably followed the administration of morphia.

Dr. John Blake White (*Practitioner*, Nov., p. 379) says that it not only relieves the pain in headaches from whatever causes, but it possesses reliable prophylactic virtues against recurrent attacks of cranial neuralgia.

Dr. Germain See (*Therapeutic Gazette*) says: "Antipyrine is the remedy par excellence for pain. He reports four cases of facial neuralgia—one of which was inveterate; six of migraine (one failure); five cases of sciatica (one failure); five cases of locomotor ataxia, in four of which the pain was controlled with antipyrine; in six cases of cardiac disease and three of aneurism, the painful manifestations disappeared without any evil effects being caused on the strength or regularity of the pulsations. The same author also reports favorably on its use, hypodermically in biliary calculi and renal colic, in asthma and severe dispnœas, from whatever cause. In these cases it relieves the pain and spasm without in any way unfavorably modifying the secretions.

T. S. Roberson has reported (*Medical Record*, May, 1887) eighty cases of migraine treated with antipyrine. Fifty-four of these were promptly relieved; in fifteen the pain was much abated and in eight it had no effect.

Dr. T. Mortimer Lloyd (*Gailard's Journal*) Feb., 1888) reports two cases of sciatica and one of intercostal neuralgia promptly relieved by hypodermic injections of antipyrine. *In one* of severe lumbago and sciatica, in which morphia gave but temporary relief, antipyrine gave more prolonged relief, and, being continued twice daily, kept the patient quite comfortable and soon put him upon his feet. He gave 7½ grains dissolved in a teaspoonful of water, but the solution was too concentrated and produced some inflammation at seat of puncture, and he recommends that it be further diluted.

I might multiply instances in which good results have been obtained from antipyrine given as an analgesic, but sufficient evidence has been adduced to show that in many cases it may be relied upon for the relief of pain, and not only does it give temporary relief, but we have many reports showing that its good effects are often permanent.

I trust many valuable suggestions may be had from this paper, and as it is already growing long I leave it to my readers to draw their own conclusions from this correlation of facts and reports. Suffice it to say that I do not hesitate to prescribe it in all cases of hyperpyrexia, and I have rarely been disappointed in its results and have not yet seen any evil effects follow its use, though I have given it in two cases where the patients were attacked with typho-malarial fever while greatly debilitated from chronic uterine disease, and the low state of the vital forces seemed to preclude the possibility of their passing through several weeks of fever and suffering; yet I had the satisfaction of seeing them both recover—retaining their strength wonderfully throughout the attack. This freedom from depression recommends the drug to our use above all other antipyretics. Given in large doses necessary to the control of temperature, quinine and salicylate of soda are notably and dangerously depressing; the cold bath is inconvenient and insufferable to a much debilitated patient, and many reports of the unpleasant after-effects of antifebrin reach us from all quarters. Antipyrine, it seems, stands alone in this freedom from all unpleasant results when given in moderate and well regulated doses. By way of warning, it may be added that two fatal cases have been reported from the use of the drug in large doses (30 to 45 grains) given to patients already much debilitated. In another instance, the liver and kidneys of a patient, to whom large doses of the drug had been given, were found to be in a state of granular and fatty metamorphosis. But these cases are rare, and much larger doses have been given where no evil effects followed.

While there is no doubt, in my mind, as to the good effects of antipyrine, both as an antipyretic and analgesic, we must not expect too much from it, for it will disappoint us many times, as will quinine, morphia and many of our standard drugs.

